# Biocompatible Hyaluronic Acid-Stabilized Copper Nanoparticles for the Selective Oxidation of Morin Dye by H_2_O_2_

**DOI:** 10.1021/acsomega.5c00769

**Published:** 2025-04-02

**Authors:** M. Deniz Yilmaz, Safaa Altves, Aliye Beyza Ozcelik, Sundus Erbas-Cakmak

**Affiliations:** † Department of Basic Sciences, Faculty of Engineering, Necmettin Erbakan University, Konya 42140, Türkiye; ‡ BITAM-Science and Technology Research and Application Center, Necmettin Erbakan University, Konya 42140, Türkiye; § Department of Molecular Biology and Genetics, Faculty of Science, Necmettin Erbakan University, 42090 Konya, Türkiye

## Abstract

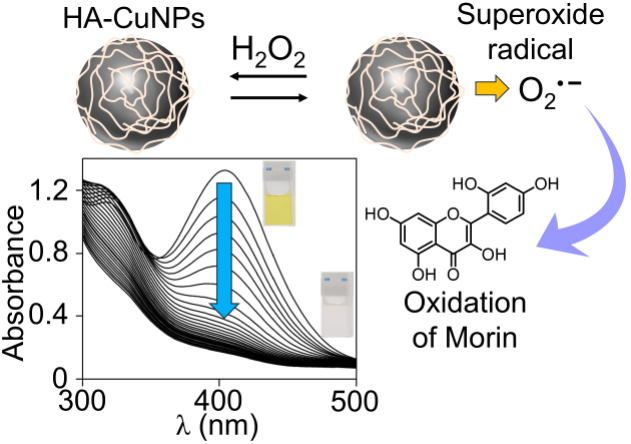

In this study, we report the synthesis and characterization of biocompatible hyaluronic acid-stabilized copper nanoparticles (HA-CuNPs) and their catalytic evaluation in the oxidation of morin as a model compound. HA-CuNPs have been characterized by several state-of-the-art analytical techniques, such as FESEM, STEM, UV–Vis, DLS, zeta potential, FTIR and XRD analyses. The average particle size and surface zeta potential of HA-CuNPs were determined to be 35 nm and −28 mV, respectively. The catalytic activity of HA-CuNPs was investigated in the oxidative degradation of morin dye in the presence of H_2_O_2_. The kinetic data show that the oxidation process follows a pseudo-first-order reaction, and the rate constant is dependent on the concentrations of morin, H_2_O_2_, and HA-CuNPs. In addition, HA-CuNPs were employed for the selective oxidation of morin on four important synthetic dyes, i.e., Congo red, methylene blue, zinc-phthalocyanine, and quinizarin. The high selectivity indicates the possible use of HA-CuNPs as low-temperature bleach catalysts for the oxidation of stains such as tea, coffee, and red wine, which contain polyphenolic compounds like morin. Further, cytotoxicity studies demonstrated the low toxicity and high biocompatibility of HA-CuNPs to Caco-2 human colorectal adenocarcinoma cells, MCF-7 human breast cancer cells, and HUVEC normal human umbilical vein endothelial cells. Combining biocompatibility with high catalytic activity could boost the potential of this eco-friendly nanocatalyst in various applications, such as wastewater treatment, laundry, textile, and wood pulp bleaching.

## 1 Introduction

Hydrogen peroxide (H_2_O_2_) bleaching, which is frequently used in the paint, textile, and paper industries, occurs under alkaline conditions (pH 9–12) at a point close to the boiling temperature.^1,2^ Such a process causes excessive energy consumption and damage to the cellulose fibers. Therefore, reducing energy consumption and protecting the cellulosic structure are becoming increasingly important for the textile industry. In this respect, the development of novel eco-friendly methods to address these issues is urgently needed.

In this direction, transition metal catalysts for the catalytic disproportionation of H_2_O_2_ came on the scene.^3−6^ The first catalysts were free transition metal cations Co^2+^, Mn^2+^, and Fe^2+^.^7^ However, the presence of free metal ions in water leads to inefficient decomposition of hydrogen peroxide and results in serious textile damage.^8,9^ To overcome these shortcomings, transition metal ions were previously formulated by combining them with sequestering agents.^10−12^ Transition metal–ligand complexes are supported to increase the stability of the metal and reduce cellulose and fiber damage. The catalytic properties of different transition metal complexes have been reported and patented so far.^13−15^ In recent years, transition metal nanoparticles in oxidative degradation of various dyes with H_2_O_2_ have attracted a great deal of attention due to their high catalytic activity, large surface area, simple preparation, low cost, and easy separation from the reaction environment.^16−28^ Among them, copper nanoparticles (CuNPs) are unique in oxidation processes because they have distinctive features such as high catalytic activity, long-term stability, small sizes, and special optical properties.^29−37^ However, CuNPs possess adverse toxicity effects on aquatic organisms and mammalian cell lines.^37−39^ The reactive oxygen species (ROS)-mediated oxidative stress is the main factor in the cytotoxic mechanism of CuNPs.^40^ Therefore, the investigation of new nontoxic, biocompatible, and effective Cu-based nanocatalysts for advanced oxidation processes is extremely critical.

**Scheme 1:**
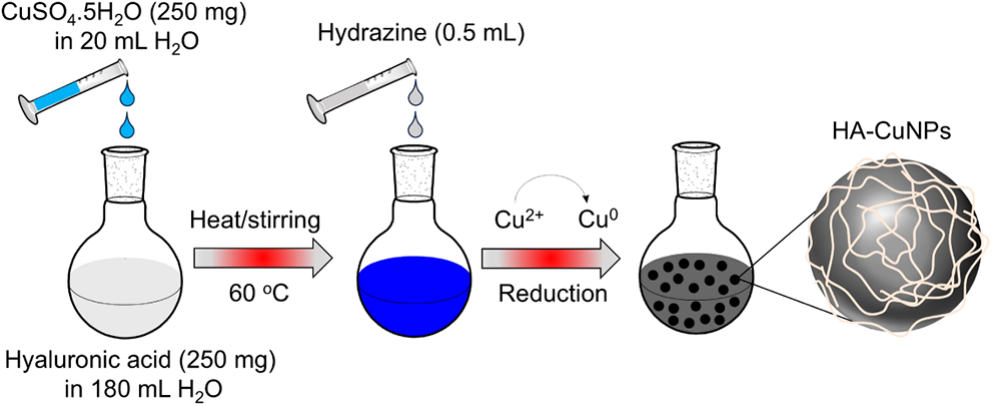
Schematic Illustration Showing the Steps of HA-CuNP Synthesis

To address these issues, we present, for the first time, hyaluronic acid (HA)-stabilized CuNPs (HA-CuNPs) as nontoxic and efficient nanocatalysts for the selective oxidation of morin with H_2_O_2_. HA is a naturally occurring polysaccharide, classified as an anionic, nonsulfated glycosaminoglycan, and it was first isolated from the vitreous of a bovine eye.^41^ It is one of the major components of the extracellular matrix, with nontoxic, biocompatible, and biodegradable characteristics.^42−46^ HA has numerous vital functions in the body, including wound repair, cell migration, and cell signaling.^47^ Owing to its high biocompatibility and biofunctionality, hyaluronic acid has played a pivotal role in biological research and has attracted a great deal of attention in several research fields, such as tissue engineering and bio(nano)medicine.^48^ HA-CuNPs were synthesized by reducing Cu^2+^ with hydrazine as the reducing agent in the presence of HA as the capping and stabilizing agent (Scheme 1). The synthesized HA-CuNPs were characterized by STEM, SEM, DLS, zeta potential, FTIR, and XRD techniques. The kinetic studies demonstrate that the oxidation process follows a *pseudo*-first-order reaction. In addition, cytotoxicity studies show the low toxicity and high biocompatibility of HA-CuNPs to Caco-2 human colorectal adenocarcinoma cells, MCF-7 human breast cancer cells, and HUVEC normal human umbilical vein endothelial cells. To our knowledge, this is the first report on the catalytic investigation of HA-CuNPs in advanced oxidation reactions.

## 2 Experimental Section

### 2.1 Materials

Unless otherwise noted, solvents and reagents were of reagent grade and used without further purification. Commercially available hyaluronic acid (low molecular weight around 30–50 kDa) (HA), morin hydrate, CuSO_4_·5H_2_O, hydrogen peroxide (30%), Na_2_CO_3_, NaHCO_3_, HCl (32%), NaOH, *t*-BuOH, NaN_3_, *p*-benzoquinone, and 3-(4,5-dimethyl-2-thiazolyl)-2,5-diphenyl-2H-tetrazolium bromide (MTT) were all purchased from Merck KGaA and used as received. Purified water (resistivity >18 MΩ·cm) was used in all experiments.

### 2.2 Preparation of HA-CuNPs

HA-stabilized CuNPs were synthesized by the reduction of Cu^2+^ to Cu^0^. Briefly, 250 mg of HA was dispersed in 180 mL of distilled H_2_O at room temperature until all the HA completely dissolved. Then, an aqueous solution of CuSO_4_·5H_2_O (250 mg in 20 mL of H_2_O) was added under vigorous stirring at room temperature. The solution was heated to 60 °C. After 30 min of mixing at 60 °C, the reducing agent hydrazine hydrate (0.5 mL) was added to the solution. The solution color immediately changed from green/blue to intense brown. The mixture was further stirred for 3 h, and H_2_O was evaporated under reduced pressure. The black solid residue was washed with acetone several times to remove impurities and dried in air for further characterization.

### 2.3 Characterization of HA-CuNPs

UV–vis measurements were conducted using a Shimadzu UV3600i Plus UV–vis-NIR spectrophotometer. Field-emission scanning electron microscopy (FESEM) and scanning transmission electron microscopy (STEM) examinations were performed on a ZEISS GeminiSEM 500 equipped with a STEM detector. DLS and zeta potential measurements were carried out using a Micromeritics Nanoplus 3 analyzer. XRD measurements were conducted using a PANalytical EMPYREAN XRD instrument. Fourier-transform infrared spectroscopy (FTIR) analyses were performed using a Thermo Scientific Nicolet iS20 system with a cycle number 60.

### 2.4 Catalytic Investigation

The catalytic reactions were carried out in a UV cuvette (1 cm path length and 2.0 mL final volume at 298 K) in 10 mM carbonate buffer at pH 10. The stock solutions of morin (1 mM in 10 mM carbonate buffer at pH 10) and HA-CuNPs (1 mg/mL in H_2_O) were freshly prepared. The appropriate amounts of reagents (200 μL of morin, 80 μL of HA-CuNPs, 100 μL of H_2_O_2_, and 1620 μL of carbonate buffer) were added into the UV cuvette to initiate the reaction, and the UV–vis spectra were collected at 1-min intervals. The following equation (eq 1) was used to calculate observed rate constants (*k*_obs_),
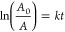
1where *A*_0_ and *A* are the absorbance values at 400 nm at 0 min and at a given time, respectively.

### 2.5 Assessment of Cell Viability

The cytotoxic effect was assessed using the 3-(4,5-dimethylthiazol-2-yl)-2,5-diphenyltetrazolium bromide (MTT) proliferation assay. Caco-2 human colorectal adenocarcinoma cells (ATCC Cat. No. HTB-37), MCF-7 human breast cancer cells, and HUVEC normal human umbilical vein endothelial cells were seeded in 96-well plates at a density of 1 × 10^5^ cells per well. After 24 h of incubation, fresh HA-CuNPs and old stored HA-CuNPs (HA-CuNPs stored in water for 3 months at ambient temperature under direct sunlight) were added at concentrations of 1, 2, 4, 8, 16, 32, 64, and 128 μg/mL. Following an additional 24 h of incubation, 10 μL of MTT reagent (5 mg/mL) was added to each well. After 4 h of incubation, the MTT solution was removed, and 100 μL of DMSO was added to solubilize the formazan crystals, followed by a 10-min incubation. Absorbance was measured at 570 nm by using a microplate reader (Multiskan SkyHigh Microplate Spectrophotometer, Thermo Scientific). Each experiment was performed in triplicate, with the control group consisting of cells with no treatment, and cell viability was calculated using the following equation (eq 2).
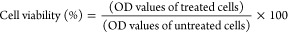
2

## 3 Results and Discussion

**Figure 1:**
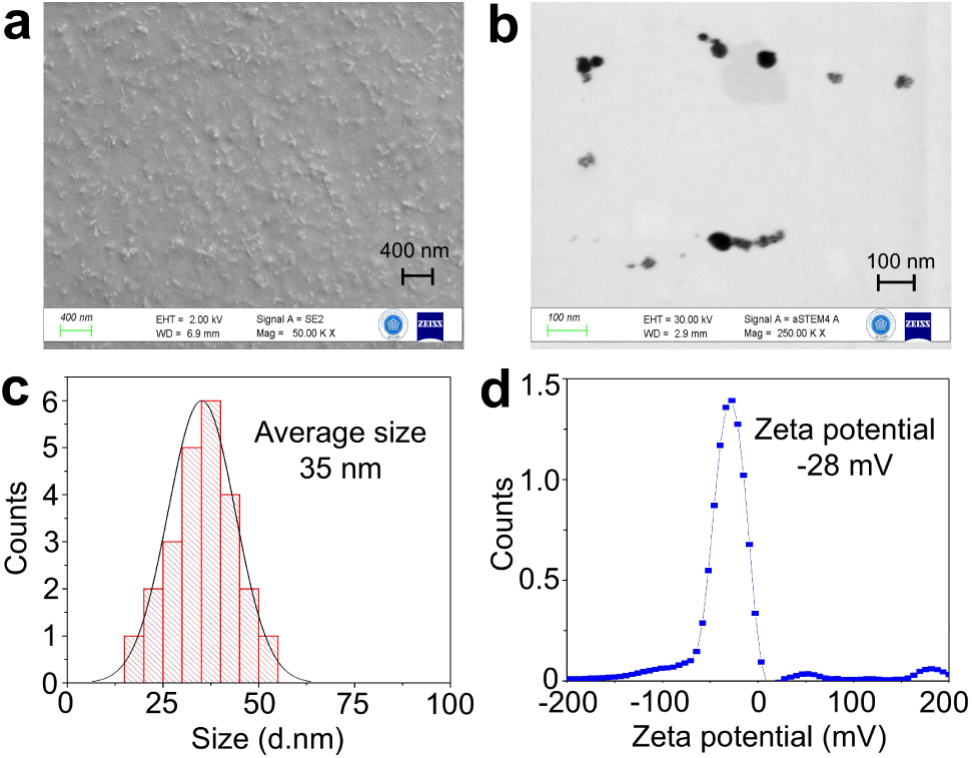
(a) Field-emission scanning electron microscopy (FESEM) image of HA-CuNPs. (b) Scanning transmission electron microscopy (STEM) image of as-synthesized HA-CuNPs. (c) Particle size distribution histogram of HA-CuNPs. (d) ζ potential of HA-CuNPs in water.

**Figure 2:**
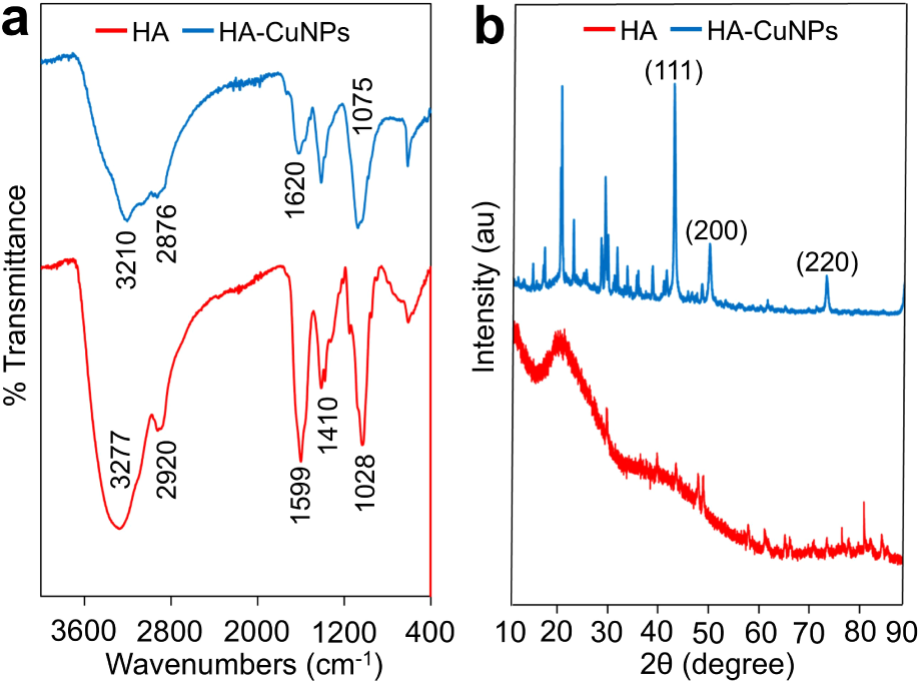
(a) FTIR spectra of HA and HA-CuNPs. (b) XRD spectra of HA-CuNPs and native HA.

HA-CuNPs were synthesized by reducing Cu^2+^ with hydrazine as a reducing agent in the presence of HA as a capping and stabilizing carbohydrate polymer (Scheme 1). The as-synthesized HA-CuNPs were characterized by several state-of-the-art FESEM, STEM, DLS, zeta potential, FTIR, and XRD analyses. FESEM (Figure 1a) and STEM (Figure 1b) images revealed that HA-CuNPs are spherical in shape and approximately 35 nm in size, as indicated by the particle size distribution histogram shown in Figure 1c. The surface zeta potential was further determined through ζ-potential measurements. In principle, nanoparticles with zeta potential values greater than ±30 mV are considered colloidally stable.^49^ The ζ-potential value of HA-CuNPs was found to be −28 mV (Figure 1d). The negative surface charge confirmed both the presence of HA on the nanoparticle surface, as the carbonyl groups of HA are deprotonated at pH 7.0, and the colloidal stability of HA-CuNPs in water via electrostatic repulsion. FTIR analyses were performed to further characterize the formation of HA-CuNPs (Figure 2a). The characteristic peaks at 3277 cm^–1^ for −OH stretching of hydroxyl groups of HA, 2920 cm^–1^ for −CH_2_ stretching of aliphatic groups of HA, 1599 cm^–1^ and 1410 cm^–1^ for the antisymmetric and symmetric stretching vibrations of COOH groups of HA, and 1028 cm^–1^ for C–O–C ester groups of HA are represented in the FTIR spectrum of pristine HA. After the formation of HA-CuNPs, the peaks at 3277 cm^–1^ and 2920 cm^–1^ shifted to lower wavenumbers due to conformational changes in HA via H-bonding and electrostatic interactions with CuNPs. On the other hand, the peaks at 1599 cm^–1^ and 1028 cm^–1^ shifted to higher wavenumbers, indicating interactions between the carboxylic groups of HA and CuNPs. The analysis results of the crystal structure of HA-CuNPs and native HA are given in Figure 2b. The presence of 2θ diffraction peaks at 43.3°, 50.4°, and 74.1° corresponding to (111), (200), and (220) planes of Cu (JCPDS file No. 89–2838 and JCPDS file No. 85-1326)^50^ clearly demonstrates the crystalline structure of HA-stabilized Cu particles. On the other hand, the peaks with low intensities around 20°–40° 2θ may be attributed to partially oxidized Cu like CuO.^51−53^ When compared with the XRD data of HA-CuNPs, the XRD spectrum of native HA showed a broad peak observed at 21°, indicating the amorphous nature of HA.

**Figure 3:**
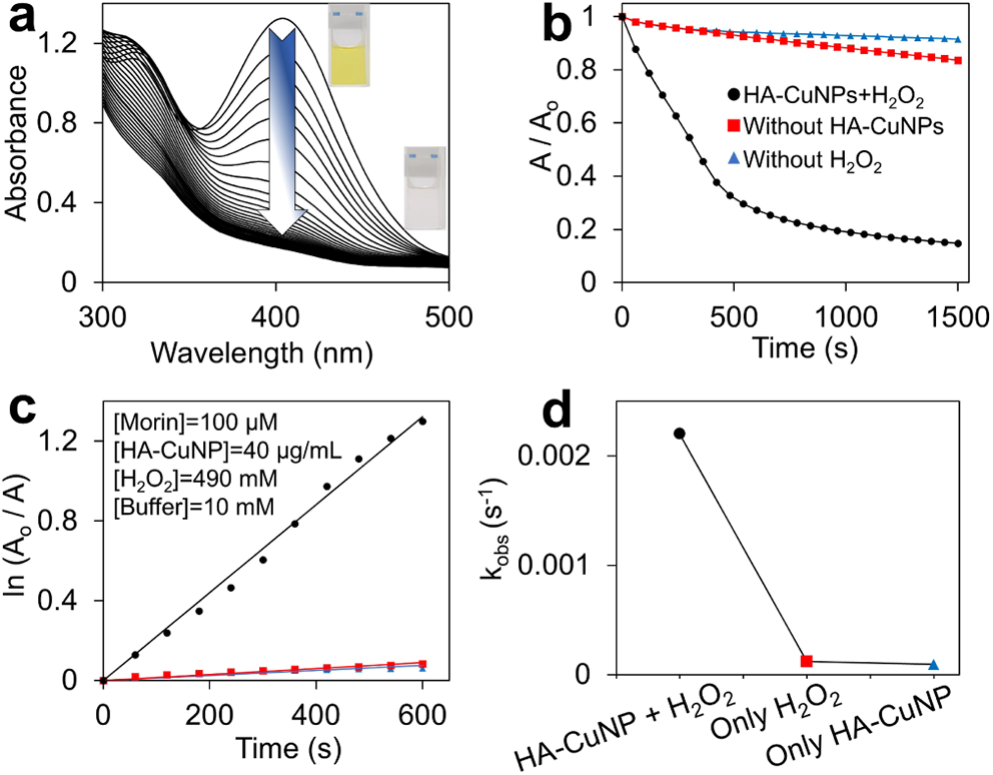
(a) Change in the UV–Vis spectra of morin in the presence of HA-CuNPs and H_2_O_2_. [Morin] = 0.1 mM, [H_2_O_2_] = 490 mM, [HA-CuNPs] = 40 μg/mL, and [Buffer] = 10 mM carbonate buffer at pH 10 at 298 K. (b) The kinetic plots of the oxidation of morin and (c) the first-order kinetic plots with and without HA-CuNPs. (d) Effect of HA-CuNPs on the *k*_obs_ values.

The application of HA-CuNPs was studied for the oxidative decolorization of morin dye in water with the help of H_2_O_2_ at room temperature. Morin is a naturally occurring flavonol that is commonly used for dyeing cotton and jute fabrics.^54^ For this reason, it has been considered and frequently used as an ideal compound for the exploration of the catalytic activity of transition metal catalysts.^55^ The absorption spectrum of morin in the presence of both HA-CuNPs and H_2_O_2_ was monitored at specific reaction intervals. As depicted in Figure 3a, the characteristic absorbance peak of morin, located at 400 nm and ascribed to the π–π* transition, decreased over time, and the solution color changed from yellow to colorless within 30 min. To investigate the catalytic effect of HA-CuNPs, the UV–vis experiment was repeated without the catalyst and H_2_O_2_ (Figure 3b). In the absence of HA-CuNPs and H_2_O_2_, absorbance changes were negligible, indicating that the coexistence of HA-CuNPs and H_2_O_2_ is essential for the efficient oxidation of morin dye. The logarithmic function of absorbance changes of morin (ln *A*_0_/*A*) was plotted against time, and the results showed that the oxidation process follows *pseudo*-first-order reaction. The observed rate constants (*k*_obs_) were determined by using eq 1. The results were calculated as follows: 2.2 × 10^–3^ s^–1^ with HA-CuNPs and H_2_O_2_, 1.2 × 10^–4^ s^–1^ with only H_2_O_2_, and 9.1 × 10^–5^ s^–1^ with only HA-CuNPs.

**Figure 4:**
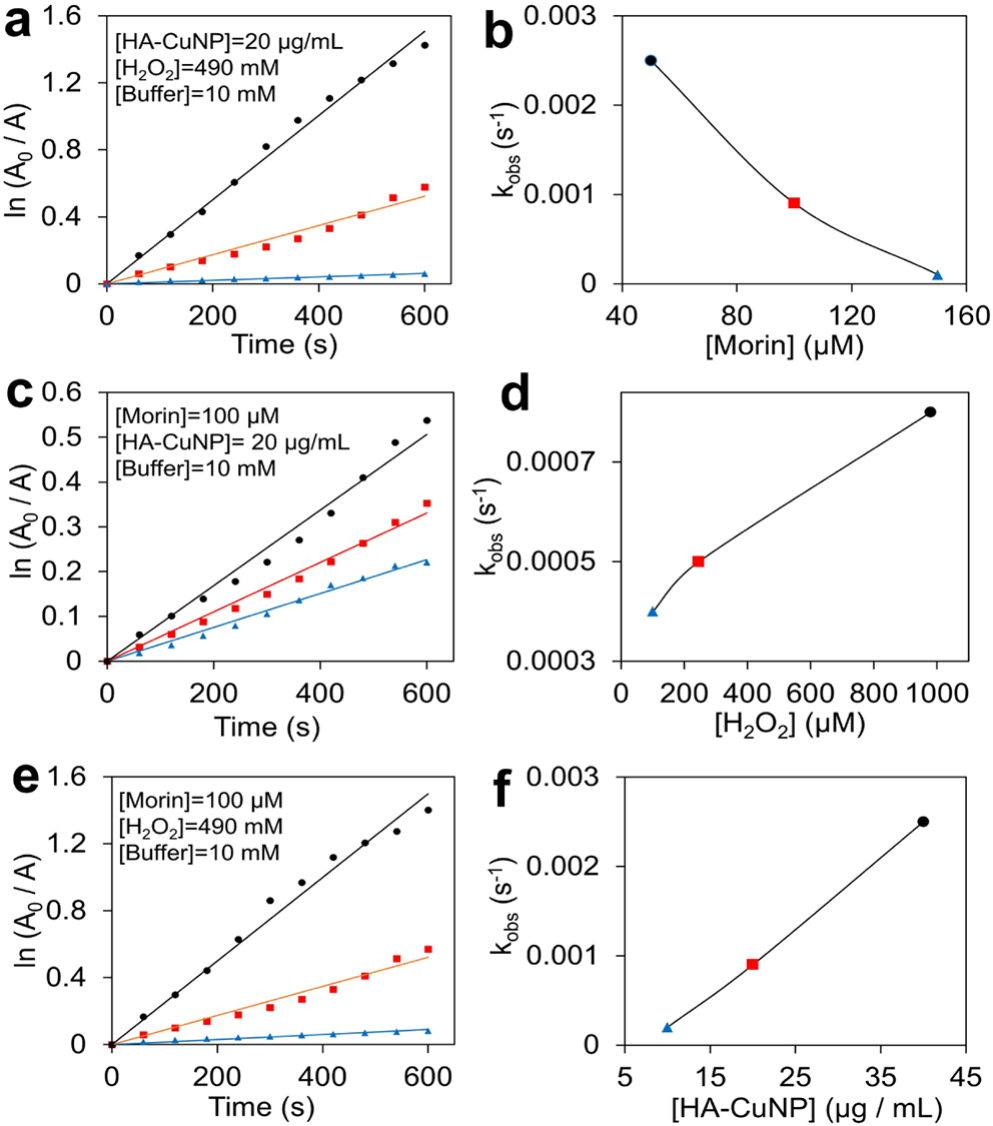
(a) First-order kinetic plots of different concentrations of morin (●, 50 μM; ■,100 μM; ▲, 150 μM) at constant concentrations of HA-CuNPs (20 μg/mL) and H_2_O_2_ (490 mM) at 298 K. (b) The plot of *k*_obs_ values versus morin concentration. (c) First-order kinetic plots of different concentrations of H_2_O_2_ (●, 980 mM; ■, 245 mM; ▲, 100 mM) at constant concentrations of HA-CuNPs (20 μg/mL) and morin (0.1 mM) at 298 K. (d) The plot of *k*_obs_ values versus H_2_O_2_ concentration. (e) First-order kinetic plots of different concentrations of HA-CuNPs (●, 40 μg/mL; ■, 20 μg/mL; ▲, 10 μg/mL) at constant concentrations of H_2_O_2_ (490 mM) and morin (0.1 mM) at 298 K. (f) The plot of *k*_obs_ values versus HA-CuNPconcentration.

The effects of the concentrations of morin, HA-CuNPs, and H_2_O_2_ on *k*_obs_ values were further investigated to obtain more details about the catalytic properties of HA-CuNPs. By tracking the absorbance changes of different concentrations of morin in the presence of constant concentrations of HA-CuNPs and H_2_O_2_, ln(*A*_0_/*A*) values were plotted against time (Figure 4a) and *k*_obs_ values were calculated as 2.5 × 10^–3^ s^–1^ for 50 μM morin, 9.0 × 10^–4^ s^–1^ for 100 μM morin, and 1.0 × 10^–4^ s^–1^ for 150 μM morin, respectively. Figure 4b shows that *k*_obs_ decreases with increasing morin concentration. These results indicate that morin adsorbs onto the catalyst and occupies the active sites of the catalyst surface, thereby reducing the overall catalytic activity.

Conversely, *k*_obs_ values increase with increasing H_2_O_2_ concentration (Figures 4c,d). We therefore conclude that both morin and H_2_O_2_ competitively adsorb onto the catalyst surface. Similarly, *k*_obs_ increases linearly with increasing HA-CuNP concentration (Figure 4e,f). This linear relationship indicates that the catalytically active sites of HA-CuNPs increase and speed up the catalytic reaction. In summary of the kinetic studies, catalytic oxidation occurs on the surface of HA-CuNPs, and the rate of the reaction depends on the concentration of morin, catalyst (HA-CuNPs), and oxidant (H_2_O_2_).

**Figure 5:**
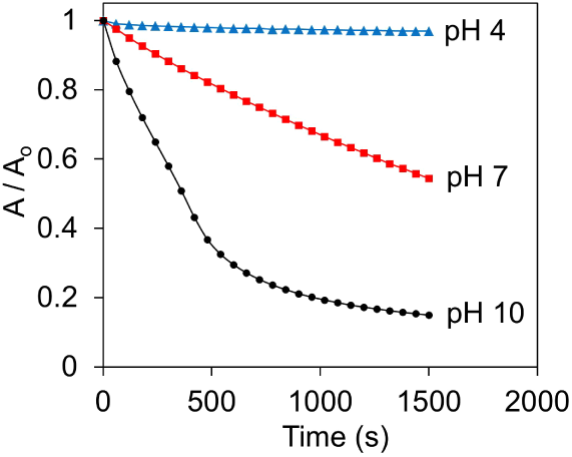
Kinetic plots of the oxidation of morin (0.1 mM) in the presence of H_2_O_2_ (490 μM) and HA-CuNPs (40 μg/mL) at different pH values at 298 K.

The effect of pH on the catalytic oxidation of morin in the presence of HA-CuNPs and H_2_O_2_ was further investigated to determine the optimum experimental conditions. At different pH values, the absorbance changes of morin at 400 nm were monitored, and the results were plotted against time (Figure 5). The results showed that the effective disproportionation of H_2_O_2_ was obtained at basic pH values, whereas the disproportionation rate was slowed when the solution pH was neutral or acidic. This result could be explained by the fact that H_2_O_2_ dissociates into perhydroxyl ion (HOO^–^) at basic pH, that will then react with HA-CuNPs to generate ROS such as superoxide (O_2_^•–^) and hydroxyl (•OH). At lower pH values, the formation of HOO^–^ is prevented; therefore, the decomposition of H_2_O_2_ is greatly favored at basic pH values.

**Figure 6:**
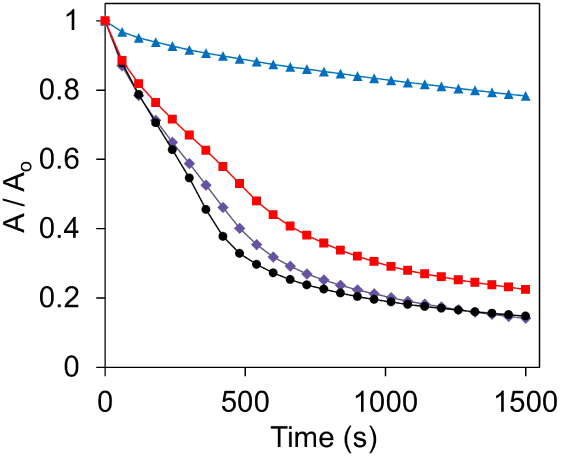
Kinetic plots depicting the oxidation of morin (0.1 mM) in the presence of HA-CuNPs (40 μg/mL) and H_2_O_2_ (490 mM) in 10 mM carbonate buffer at pH 10 at 298 K without any radical scavengers (●), with 100 mM *t*-BuOH (⧫), 100 mM NaN_3_ (■), and 100 mM *p*-benzoquinone (▲).

To examine the mechanism of catalytic action of HA-CuNPs, radical scavengers such as NaN_3_ for singlet oxygen (^1^O_2_), *p*-benzoquinone for superoxide (O_2_^•–^), and *t*-butanol for hydroxyl radicals (•OH) were added into the reaction environment, and the absorbance changes of morin at 400 nm were plotted against time. As can be seen from Figure 6, the absorbance change was negligible when *p*-benzoquinone was utilized as a radical scavenger; however, a drastic change was observed by NaN_3_ and *t*-butanol. The results clearly indicate that superoxide (O_2_^•–^) is the main radical species in the catalytic oxidation of morin by HA-CuNPs.

**Scheme 2:**
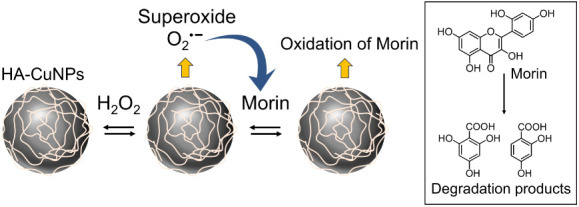
Proposed Mechanism of Catalytic Oxidation of Morin using HA-CuNPs in the Presence of H_2_O_2_

The results summarized in Figure 4 demonstrate that *k*_obs_ decreases with an increasing concentration of morin and increases with an increasing H_2_O_2_ concentration. Obviously, this reaction is not controlled by the diffusion of morin and H_2_O_2_ on the HA-CuNP surface. Based on these observations and the results we obtained, the catalytic oxidation follows the Langmuir–Hinshelwood model (Scheme 2).^21,27^ This model assumes that H_2_O_2_ molecules initially adsorb reversibly onto the surface of HA-CuNPs. HA-CuNPs catalyze H_2_O_2_ to yield a compound called O_2_^•–^. Concomitantly, morin dyes adsorb onto the free sites of HA-CuNPs. The reaction between O_2_^•–^ and the adsorbed morin on the surface is the rate-determining step for this catalytic process. Finally, the degradation products desorb from the surface, and the newly available active sites are ready for the next catalytic cycle.^26,56^

**Scheme 3:**
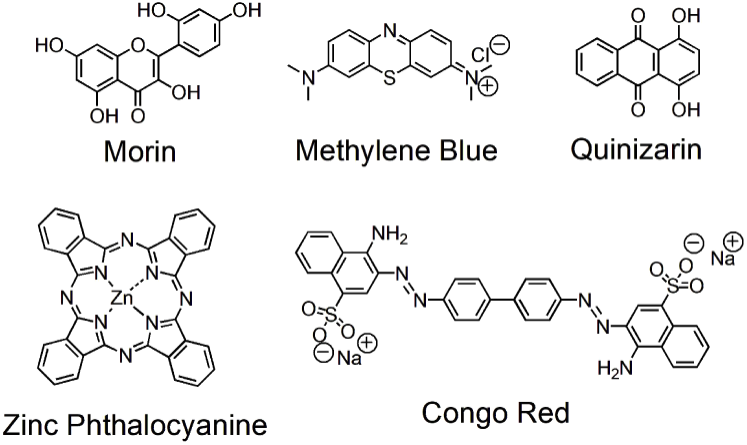
Chemical Structures of Dyes Used in this Study

**Figure 7:**
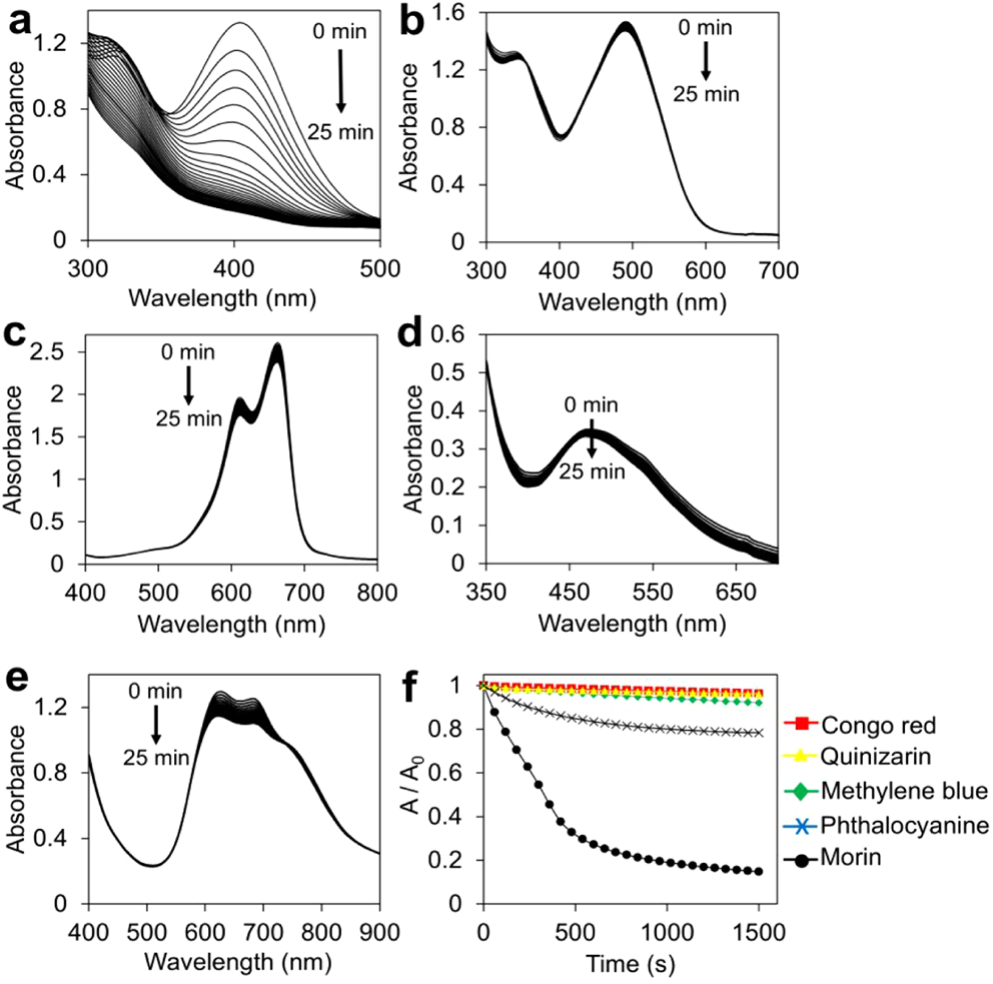
(a) Change in the UV–vis spectrum of morin. [Morin] = 0.1 mM. (b) Change in the UV–vis spectrum of congo red. [Congo red] = 0.1 mM. (c) Change in the UV–vis spectrum of methylene blue. [Methylene blue] = 0.05 mM. (d) Change in the UV–vis spectrum of quinizarin. [Quinizarin] = 0.05 mM. (e) Change in the UV–vis spectrum of zinc phthalocyanine. [Zn phthalocyanine] = 0.1 mM (f) The kinetic plots of the oxidation of dyes in the presence of HA-CuNPs and H_2_O_2_. [H_2_O_2_] = 490 mM, [HA-CuNPs] = 40 μg/mL, and [Buffer] = 10 mM carbonate buffer at pH 10 at 298 K.

To investigate the selective oxidation of morin dye over representative examples of synthetic textile dyes, i.e., Congo red (azo dye), methylene blue (xanthene dye), phthalocyanine (cyanine dye), and quinizarin (anthraquinone dye) (Scheme 3),^57^ the absorbance changes were monitored at different time intervals in the presence of constant concentrations of HA-CuNPs and H_2_O_2_. The absorbance peak of morin decreased over time (Figure 7a); however, the absorbance peaks of Congo red (Figure 7b), methylene blue (Figure 7c), quinizarin (Figure 7d), and phthalocyanine (Figure 7e) remained unchanged. These results may be attributed to the free radical scavenging capacity of morin dye, which contains five hydroxyl (−OH) groups in its chemical structure, hence increasing the free radical scavenging effects compared to the chemical structures of other textile dyes that do not have abundant hydrogen donors in their chemical structure (Scheme 3).^58−60^ As a result, morin dye was selectively oxidized by HA-CuNPs, which can be used as oxidation catalysts for the removal of fabric stains, such as tea, coffee, and red wine, which contain polyphenols like morin, while protecting the synthetic textile dyes in bleaching processes.

**Figure 8:**
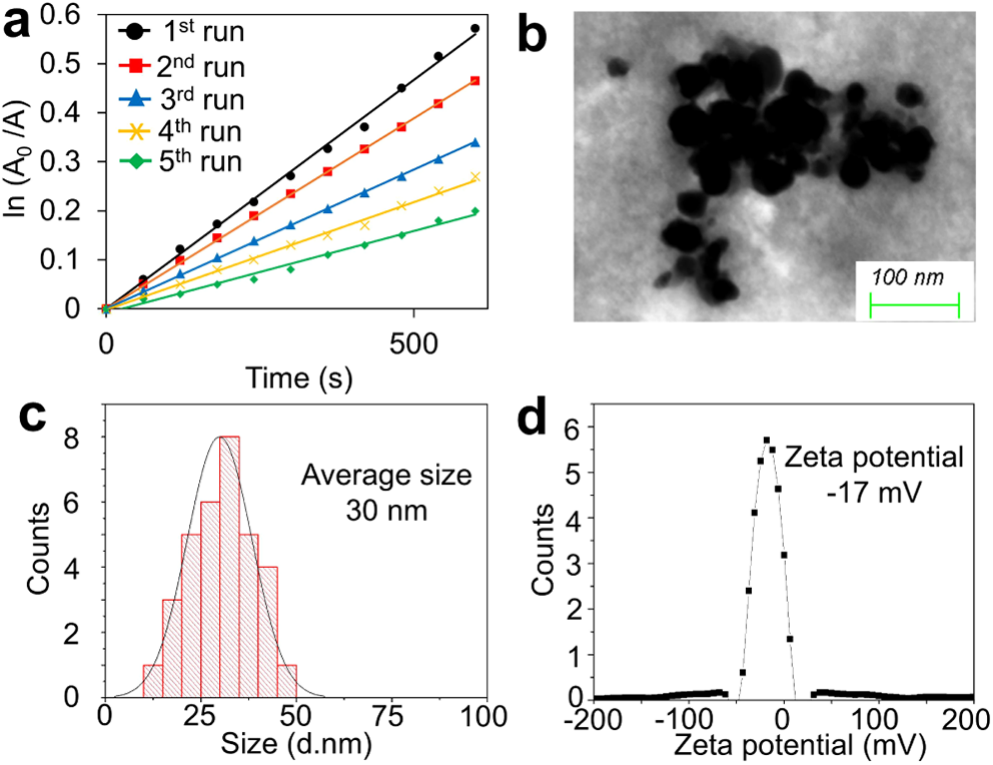
(a) First-order kinetic plots of the oxidation of morin for the five consequent catalytic runs. [Morin] = 0.1 mM, [H_2_O_2_] = 490 mM, [HA-CuNPs] = 20 μg/mL, and [Buffer] = 10 mM carbonate buffer at pH 10 at 298 K. (b) STEM image of recycled HA-CuNPs. (c) Particle size distribution histogram of recycled HA-CuNPs. (d) ζ-potential of recycled HA-CuNPs in water.

To examine the reusability and recyclability of HA-CuNPs, the oxidation of morin with HA-CuNPs over five cycles was repeated. The *k*_obs_ values for each cycle were calculated following eq 1 and were found to be 9.0 × 10^–4^ s^–1^ for the first run, 8.0 × 10^–4^ s^–1^ for the second run, 6.0 × 10^–4^ s^–1^ for the third run, 4.0 × 10^–4^ s^–1^ for the fourth run, and 3.0 × 10^–4^ s^–1^ for the fifth run. The rate decreased consistently after the five cycles, as shown in Figure 8a. The spherical structure of recycled HA-CuNPs remained unaltered; however, some degree of aggregation was observed (Figure 8b) indicating a certain level of colloidal instability of HA-CuNPs after repetitive use. According to the particle size distribution histogram, the size of HA-CuNPs was approximately 30 nm, demonstrating that the average size remained unchanged after the catalytic processes (Figure 8c). Furthermore, the surface zeta potential was found to be −17 mV, proving the presence of HA on the nanoparticle surface after the five subsequent runs (Figure 8d). These results demonstrate that HA-CuNPs can be recycled and reused multiple times in oxidation processes.

**Figure 9:**
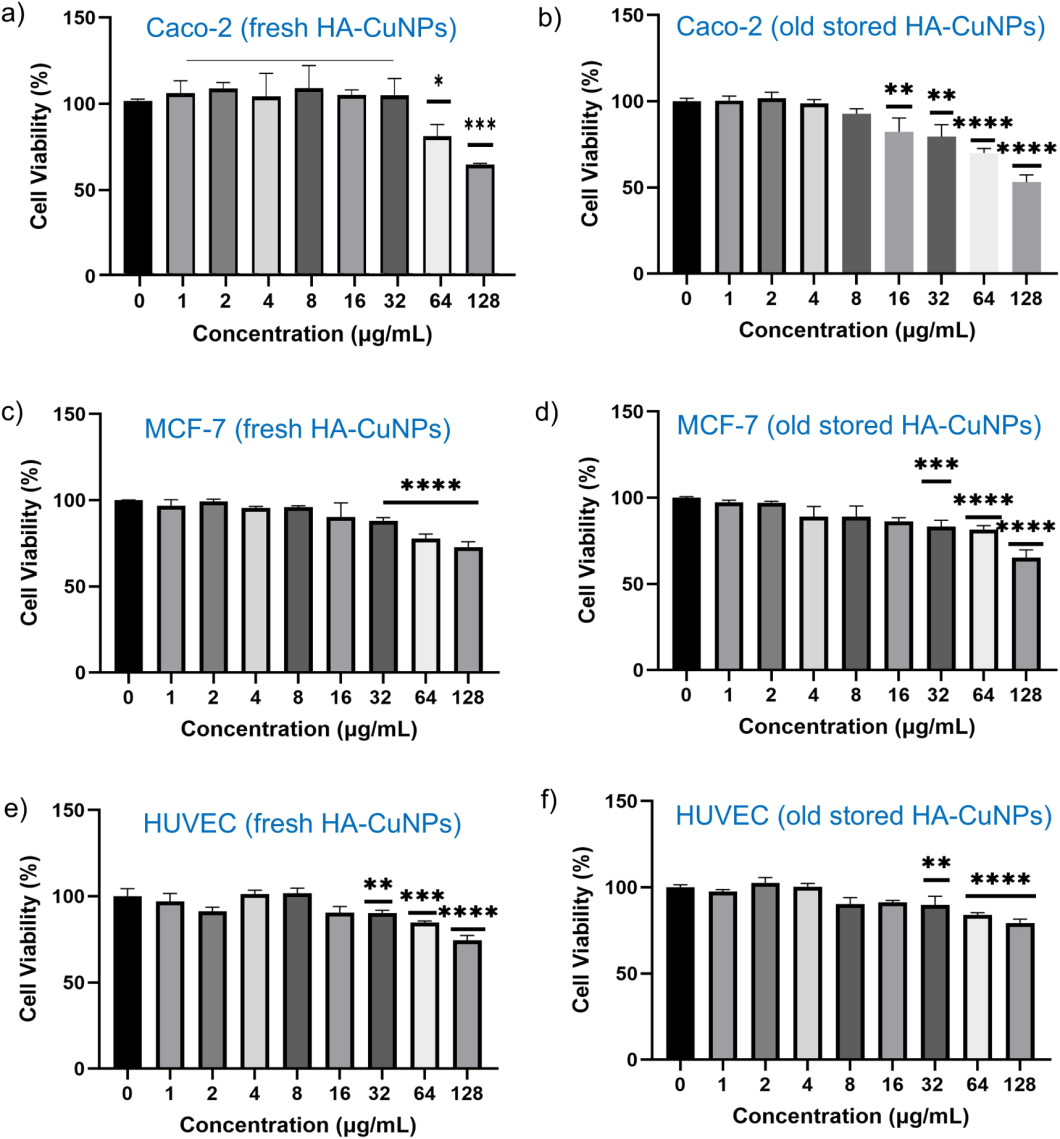
Viability of (a, b) Caco-2 human colorectal adenocarcinoma Cells (ATCC Cat. No. HTB-37), (c, d) MCF-7 human breast cancer cells, and (e, f) HUVEC normal human umbilical vein endothelial cells incubated with various concentrations of fresh (a, c, e) and old stored (b, d, f) HA-CuNPs (*n* ≥ 3, *p ≤ 0.05; **p ≤ 0.01; ***p ≤ 0.001; ****p ≤ 0.0001).

Toxic effects of copper nanoparticles (CuNPs) in both plant and animal species have been well studied and demonstrated in the literature.^40^ The main mechanism of toxicity is generally related to ROS generation and oxidative stress induction in living cells. To address this issue, biocompatible and nontoxic HA was applied as a capping and stabilizing agent to reduce the toxic effects of CuNPs. The cytotoxic effects of fresh HA-CuNPs and old stored HA-CuNPs (HA-CuNPs stored in water for 3 months at ambient temperature under direct sunlight) were assessed using the 3-(4,5-dimethylthiazol-2-yl)-2,5-diphenyltetrazolium bromide (MTT) proliferation assay. The concentration-dependent cell viability results are summarized in Figure 9. The results clearly demonstrate that, for all cells, viability is preserved at or above 80% when long-storage or fresh samples are applied up to 32 μM. This value is much higher than the catalyst concentration that was employed in the oxidation studies. As a result, HA-CuNPs or any byproducts generated during long exposure are not toxic to cells derived from different tissues, including healthy HUVEC cells.

## 4 Conclusions

In summary, the preparation and characterization of hyaluronic acid-capped copper nanoparticles (HA-CuNPs) and their catalytic evaluation on the oxidation of morin as a model compound were reported. The average particle size and surface zeta potential of HA-CuNPs were determined by DLS and zeta potential measurements and found to be 35 nm and −28 mV, respectively. The negative surface charge was attributed to the carboxylic acid moieties of HA. The kinetic studies of the catalytic reaction between morin and HA-CuNPs in the presence of H_2_O_2_ showed that the oxidation reaction followed a *pseudo*-first-order reaction. The observed rate constants (*k*_obs_) were calculated, and the results proved that rate constant values increased with increasing HA-CuNPs and H_2_O_2_ concentrations, whereas *k*_obs_ decreased with increasing concentration of morin. In addition, the selective oxidation of morin over other synthetic dyes, such as Congo red, methylene blue, zinc phthalocyanine, and quinizarin, was observed. Low cell toxicity and high catalytic activity with interesting selectivity make HA-CuNPs a promising candidate for various applications such as wastewater treatment, laundry, textile, and wood pulp bleaching. To this end, further studies are ongoing in our laboratory.
